# Rapid Laser Manufacturing of Microfluidic Devices from Glass Substrates

**DOI:** 10.3390/mi9080409

**Published:** 2018-08-17

**Authors:** Krystian L. Wlodarczyk, Richard M. Carter, Amir Jahanbakhsh, Amiel A. Lopes, Mark D. Mackenzie, Robert R. J. Maier, Duncan P. Hand, M. Mercedes Maroto-Valer

**Affiliations:** 1 Research Centre for Carbon Solutions (RCCS), Institute of Mechanical, Process and Energy Engineering, School of Engineering and Physical Sciences, Heriot-Watt University, Edinburgh, EH14 4AS, UK; A.Jahanbakhsh@hw.ac.uk (A.J.); M.Maroto-Valer@hw.ac.uk (M.M.M.-V.); 2 Institute of Photonics and Quantum Sciences, School of Engineering and Physical Sciences, Heriot-Watt University, Edinburgh, EH14 4AS, UK; R.M.Carter@hw.ac.uk (R.M.C.); aal1@hw.ac.uk (A.A.L.); M.Mackenzie@hw.ac.uk (M.D.M.); R.R.J.Maier@hw.ac.uk (R.R.J.M.); D.P.Hand@hw.ac.uk (D.P.H.)

**Keywords:** microfluidic devices, laser materials processing, ultrafast laser micromachining, ultrafast laser welding, enclosed microstructures, glass, porous media, fluid displacement

## Abstract

Conventional manufacturing of microfluidic devices from glass substrates is a complex, multi-step process that involves different fabrication techniques and tools. Hence, it is time-consuming and expensive, in particular for the prototyping of microfluidic devices in low quantities. This article describes a laser-based process that enables the rapid manufacturing of enclosed micro-structures by laser micromachining and microwelding of two 1.1-mm-thick borosilicate glass plates. The fabrication process was carried out only with a picosecond laser (Trumpf TruMicro 5×50) that was used for: (a) the generation of microfluidic patterns on glass, (b) the drilling of inlet/outlet ports into the material, and (c) the bonding of two glass plates together in order to enclose the laser-generated microstructures. Using this manufacturing approach, a fully-functional microfluidic device can be fabricated in less than two hours. Initial fluid flow experiments proved that the laser-generated microstructures are completely sealed; thus, they show a potential use in many industrial and scientific areas. This includes geological and petroleum engineering research, where such microfluidic devices can be used to investigate single-phase and multi-phase flow of various fluids (such as brine, oil, and CO_2_) in porous media.

## 1. Introduction

Microfluidic devices are used across a wide range of applications in many industrial and research areas, primarily in chemistry, biology, medicine, and pharmacology [1,2,3,4,5,6,7,8,9]. These devices enable the direct observation and investigation of various physical, chemical, and biological processes occurring at small (even sub-micron) length scales. An operation on such small volumes obviously reduces the amount of testing materials (such as fluids, colloids, cells, etc.) and the time required for analysis, thereby reducing the overall cost of an experiment. Many microfluidic devices also offer temperature control and parallel operation. Simultaneous operations can be executed thanks to the compact size of microfluidic devices. Finally, the hermeticity of microfluidic devices reduces the risk of sample contamination and provides a physical barrier between an operator and an analyzed substance that sometimes can be dangerous (e.g., living cells and bacteria).

Microfluidic devices are also used in geological and petroleum engineering research to investigate various processes governing the macroscopic behavior of subsurface systems at a pore level (i.e., sub-micron scale) [10,11]. These microfluidic devices, often called “micromodels”, typically contain structures of interconnected pores whose arrangement and shapes are designed in such a way to represent simplified versions of the geometries typically found in the subsurface systems. In other words, they are constructed to mimic, as close as possible, an internal structure of rocks. Using such microfluidic devices, it is possible to observe and study processes, such as CO_2_ injection and trapping [12,13,14,15,16], oil recovery [17,18,19,20], dissolution of substances [21], and transport of colloids [22], at a’ small (sub-millimeter) scale in the laboratory environment.

Microfluidic devices can be manufactured from a range of materials, such as SU-8 photoresist, polydimethylsiloxane (PDMS), polymethyl methacrylate (PMMA), polylactic acid (PLA), cyclic olefin copolymer (COC), glass, or silicon. Glass, due to its unique combination of high transparency, hardness, thermal stability, electric insulation, surface stability, chemical inertness, and resistance to acids, is often a preferred substrate for the fabrication of microfluidic devices over silicon and polymers. Unfortunately, the conventional manufacturing of microfluidic devices from glass substrates is a complex, multi-step process that involves different fabrication techniques and tools [23,24,25]. This, in turn, makes the fabrication process time-consuming and expensive, in particular for the prototyping of microfluidic devices in low quantities.

Microfluidic patterns on glass materials are typically generated by either wet (chemical) etching or dry (reactive ion) etching. Wet etching of glass involves the use of strong chemicals, such as hydrofluoric acid (HF), to remove the material [24]. This etching technique enables the manufacturing of deep structures (>500 µm) with an etch rate of several µm/min. The surface roughness of the etched structures can be as low as 10 nm. Unfortunately, the etched structures have a low aspect ratio (close to unity) because this process is isotropic. This means that such structures contain walls with rounded corners and may possess undercuts.

The reactive ion etching (RIE) of glass is an anisotropic process that enables the generation of microfluidic patterns with almost vertical sidewalls (the wall angles up to 88°) [25]. Microchannels generated by RIE can have a very high aspect ratio (up to 40) and low surface roughness (Ra <10 nm). The drawbacks of RIE are a low etching rate (typically <1 µm/min) and a low etch selectivity that forces the use of thick masks. This, in turn, reduces the spatial resolution of etching structures.

Although both etching techniques enable the precision generation of complex microfluidic patterns on glass substrates [24,25], the generation of these patterns is limited to only two dimensions. Moreover, these two fabrication techniques require the preparation of bespoke masks made of either a photoresist or metal. Since such masks are manufactured by photolithography and etching, the entire fabrication process of microfluidic devices is time-consuming and can be expensive, particularly at the prototyping stage.

Lasers enable the generation of microfluidic patterns on various materials, including glass materials [25,26,27,28,29,30,31,32]. Direct (maskless) writing of microstructures on the surface of glass can be obtained using a CO_2_ laser, an ultrafast laser, an excimer laser, or an ultraviolet Q-switched solid-state laser. Current laser systems, which often are integrated with a galvo-scanning system and computer-aided design (CAD) software, enable the generation of complex 2.5-dimensional (2.5D) patterns in which each channel may have a different depth and width. The surface elements of such patterns may be generated with different sets of laser parameters, and thus, they may have different widths as well as depths.

Ultrafast (femtosecond) lasers can also be used for the generation of truly three-dimensional (3D) microfluidic patterns inside glass materials, such as fused silica, Borofloat^®^33, or Pyrex™ [33,34,35,36,37,38]. This is performed by chemically etching the locally laser-modified regions inside glass. This fabrication technique, often called selective laser-induced etching (SLE), enables the manufacturing of microfluidic devices without the use of a physical mask and additional steps related to the enclosure of the microfluidic patterns. The recent results presented by Gottmann et al. [38] provided strong evidence that the SLE process is an attractive alternative to the conventional etching processes for the fabrication of glass microfluidic devices. Unfortunately, the drawback of this process is a very long etching time (even a few days to complete the development of a microstructure).

In the past, we demonstrated that an ultrafast picosecond laser (Trumpf TruMicro 5×50, Trumpf Ltd., Ditzingen, Germany) can be an effective tool for the direct cutting, drilling, and micromachining of glass plates [39,40], and for joining glass to glass [41,42] or even glass to metal [41] without using any intermediate adhesive layers. In this article, we report on a combination of these processes to manufacture glass microfluidic devices. A picosecond laser was used to generate microfluidic patterns directly on glass (using laser ablation) and to enclose such patterns by microwelding a cover glass plate. Inlet/outlet ports in the cover glass were also generated with the same laser. This process provides a high degree of flexibility in the design of microfluidic devices, which is very important, particularly at the stage of prototyping, and reduces the time and cost associated with their manufacture when a low quantity of the devices is required.

## 2. Materials and Methods

### 2.1. Material Used

Borosilicate (Schott Borofloat^®^33) glass plates with dimensions of 75 mm × 25 mm × 1.1 mm were used for the manufacturing of microfluidic devices. The flatness of the glass plates was ~λ/4. Borofloat^®^33 contains 81% SiO_2_, 13% B_2_O_3_, 4% Na_2_O/K_2_O, and ~2% Al_2_O_3_ [43]. This material has similar optical properties to fused silica, but it is less expensive. This glass is used in many industrial and scientific areas, such as chemistry, optics, photovoltaics, micro-electronics, and biotechnology.

### 2.2. Laser System

A customized laser processing system based around a 50-W picosecond laser (Trumpf TruMicro 5×50) was used in this work. The laser provides ~6 ps pulses (as measured at full width at half maximum (FWHM)) with a maximum pulse repetition frequency (PRF) of 400 kHz. The model TruMicro 5×50 contains three outputs; each output emits a collimated laser beam of a different wavelength (λ = 1030 nm, 515 nm, or 343 nm). The output laser beams are expanded and delivered to three separate galvo-scanners using appropriate high-reflection (HR)-coated mirrors. Each galvo-scanner is equipped with an approximately 160-mm-focal-length F-theta lens. The laser beams at the focus have different diameters and M^2^ values, as listed in Table 1.

### 2.3. Laser Micromachining Procedure

Microfluidic patterns and inlet/outlet ports were generated using the 515-nm wavelength. At this wavelength, the peak laser fluence and the machining resolution were the highest. The maximum fluence used for machining the glass plates was 31.1 J/cm^2^. The PRF value, in turn, was limited to 100 kHz because higher values could potentially lead to heat accumulation in the material, and consequently, the fusion of glass particles to the material surface [29].

**Figure 1:**
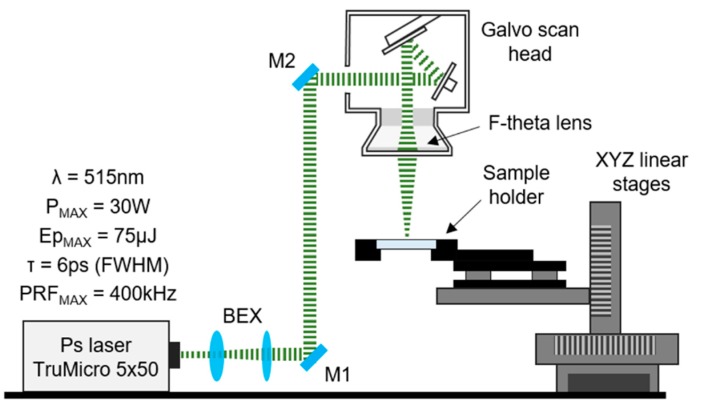
Schematic of the laser system used for machining of the glass plate.

Prior to the laser treatment, the glass samples were cleaned with isopropanol and wiped off with lens tissues. Following the cleaning process, the glass plates were mounted in a holder that provided a clear aperture underneath the laser machining area, while the holder was fixed to XYZ linear stages (Aerotech PRO115, Aerotech, Inc., Pittsburgh, PA, USA), as shown in Figure 1. The linear stages provided accurate positioning of the glass samples for machining.

### 2.4. Laser Microwelding Procedure

**Figure 2:**
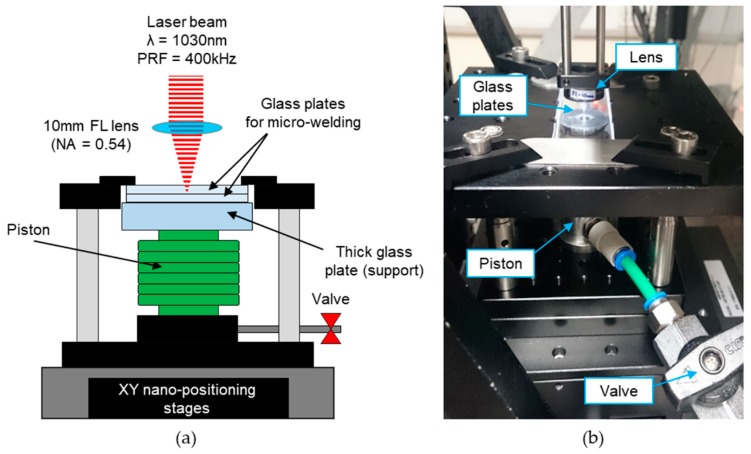
Holder used for the microwelding of two glass plates: (**a**) schematic; and (**b**) photograph.

The same picosecond laser processing system was also used for the microwelding of glass plates; however, a different processing arrangement was used. In this case the 1030-nm wavelength was used at the maximum PRF of 400 kHz. The processing arrangement was the same as that described in Reference [41]. The only modification was a holder (shown in Figure 2) which provided a large working area (70 mm × 25 mm) and allowed the glass plates to maintain close contact during the welding process. The piston underneath the glass plates, which was under pressure of 1 bar, holds the glass plates in place during the movement of the holder. A 6-mm-thick supporting glass plate prevents the 1.1-mm-thick glass plates from bending.

**Figure 3:**
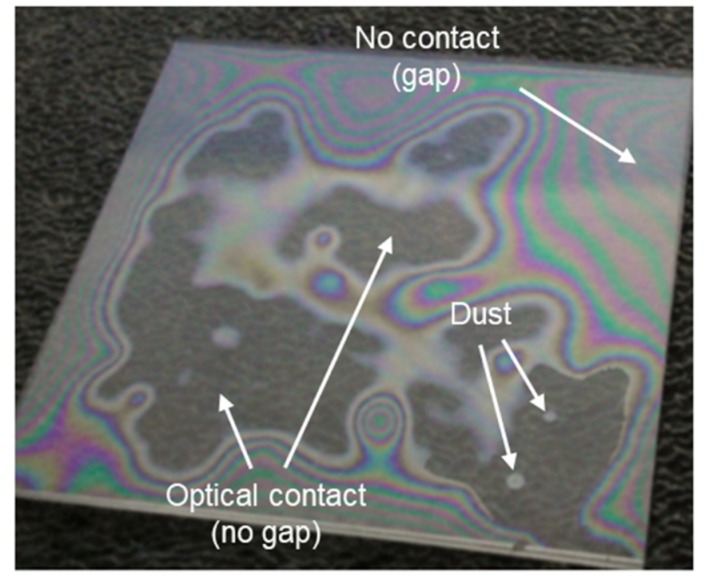
Two glass plates in local contact.

The real challenge in laser microwelding is to bring two glass plates into sufficiently close contact prior to the process. This can be achieved by pressing one plate to the other. Once optical contact is provided, van der Waals forces are capable of holding the two materials together. This, however, requires the glass surfaces to be flat, smooth, and free of debris. Figure 3 shows two glass plates in local contact. Optical fringes (so-called Newtonian rings) visible on this photo indicate a small gap between the two materials, whereas, in the areas where no optical fringes can be seen, the two glass plates can be assumed to be in optical contact.

The laser-machined glass plates were contaminated with small amounts of dust and glass particles that would prevent optical contact being achieved. Therefore, it was necessary to clean the plates prior to laser welding to ensure optical contact between the two materials. The cleaning was performed with the use of an ultrasonic bath. The glass plates were individually inserted into a beaker filled with methanol, and the beaker was placed into a water-filled tank of an ultrasonic bath, that was operated at room temperature for ~10 min. After the ultrasonic bath treatment, the samples were dried using a jet of ionized nitrogen. This last cleaning step was performed under an air hood that protected the samples from dust. The cover glass plates were cleaned in the same way.

Recently, more effective cleaning of the laser-machined samples was achieved using hydrofluoric (HF) acid. In this method, the glass plates are placed into a beaker filled with a 5% HF solution, and are kept in this solution at room temperature for a couple of minutes. This removes any debris resulting from the laser machining process, even glass particles that fused to the glass surface.

Following the cleaning process, the glass cover and the laser-machined glass plate are placed into contact, and a force is applied to bring them into optical contact. Such prepared samples are transferred to the holder, while the holder is fixed to XY nano-positioning stages (Aerotech ANT95-XY, Aerotech, Inc., Pittsburgh, PA, USA), as shown in Figure 2b. The laser spot used for microwelding had a diameter of ~3 µm in air. Such a small spot was obtained by focusing the laser beam through a 10-mm-focal-length aspheric lens. Since the laser beam was stationary, the glass samples had to move during the welding process. This was achieved by means of the XY nano-positioning stages which moved the glass plates through the fixed focus of the laser. The incident laser radiation was focused ~80 µm below the glass–glass interface in order to generate a weld seam across the interface. The procedure for the generation of welds in a specific location inside a transparent material was described in Reference [41].

### 2.5. Testing of the Laser-Manufactured Microfluidic Devices

**Figure 4:**
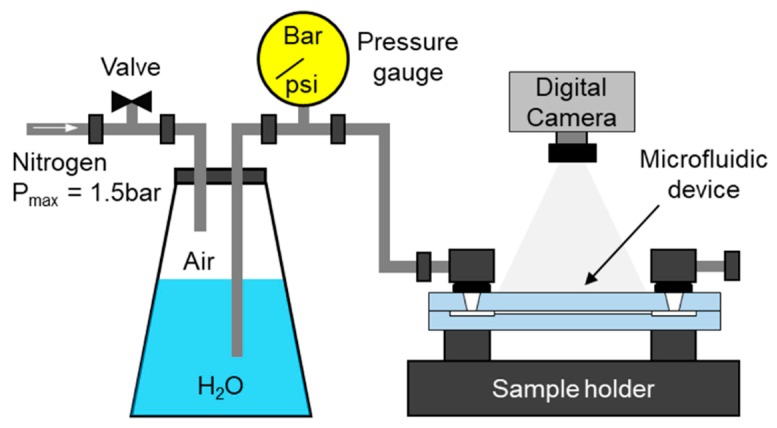
Set-up used for testing the laser-manufactured microfluidic devices.

The laser-manufactured microfluidic devices were tested using the set-up shown in Figure 4. The aim of this test was to determine whether the welds are capable of limiting the flow of fluids to the enclosed microfluidic patterns.

Initially, the microfluidic channels were filled with air. In the test, deionized (DI) water was injected into a microfluidic device through one of the inlet ports. After filling, the device was inspected for any water leakage. Following the visual inspection of the device, nitrogen was injected in order to remove the water. The maximum injection pressure was 1.5 bar. During these experiments, the flow of fluids was recorded using a digital camera (Canon IXUS 60, Canon, Inc., Tokyo, Japan).

## 3. Results and Discussion

### 3.1. Calibration of the Laser Micro-Machining Process

**Figure 5:**
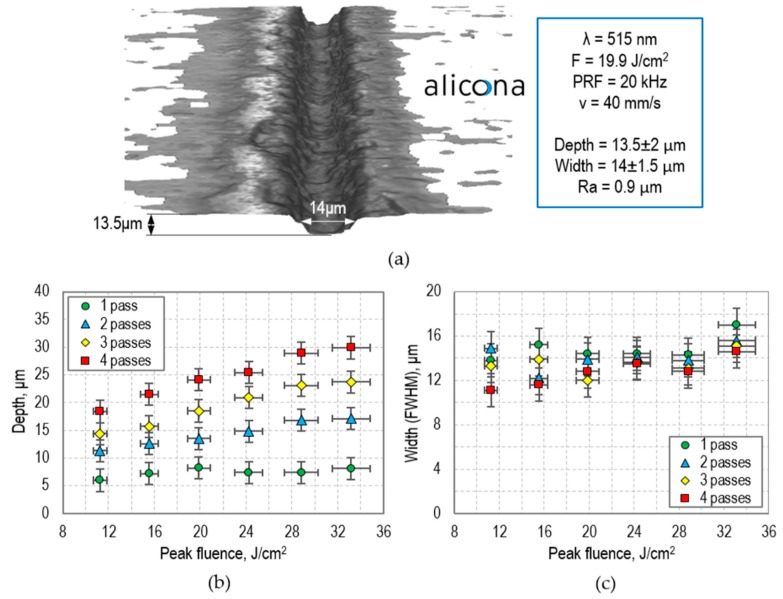
(**a**) 13.5-µm deep and 14-µm wide groove (channel) generated by the picosecond laser using a 21-µm-diameter spot; (**b**) Depth and (**c**) width (measured at full width at half maximum (FWHM)) of the channels generated with a pulse repetition frequency (PRF) of 20 kHz and a laser beam scan velocity (v) of 40 mm/s. Channels were measured using a three-dimensional (3D) surface profilometer (Alicona InfiniteFocus^®^).

Figure 5a shows that the picosecond laser (Trumpf TruMicro 5×50) can machine grooves (channels) with a roughly Gaussian cross-section. A peak laser fluence >10 J/cm^2^ (ablation threshold) is required, together with a PRF < 400 kHz, and a pulse overlap in the range of 85–95%. If the overlap is too small, the result is partial machining and the generation of damage on the back surface of glass, whereas, if the overlap and PRF are too high, the resultant heat accumulated in the material can be sufficient to cause cracking.

The laser-generated channels were measured and characterized using a 3D surface profilometer (Alicona InfiniteFocus^®^, Alicona Ltd., Raaba, Austria). This instrument can measure many surface parameters, including depth, width, and surface roughness, with a vertical resolution down to 10 nm. Figure 5a shows a groove that was generated at a peak laser fluence (F) of 19.9 J/cm^2^. The PRF value and laser beam scan velocity (v) were 20 kHz and 40 mm/s, respectively. At these laser parameters, the distance between the centers of subsequent laser pulses (Δx) was 2 µm, corresponding to a 90.4% pulse overlap (calculated as O = ((2 ω_0_ − Δx)/2 ω_0_) × 100%, where 2 ω_0_ is the laser beam diameter).

Channels with different depths can be generated by altering peak fluence and the number of laser passes, while maintaining the same velocity and PRF. As can be seen in Figure 5b, the laser-generated channels can have a minimum depth of 6 µm and a minimum width of 10 µm, as measured at FWHM. Surface roughness (Ra) along the bottom of the channels was measured to be approximately 1 µm. This value was calculated for a 0.2-mm length.

**Figure 6:**
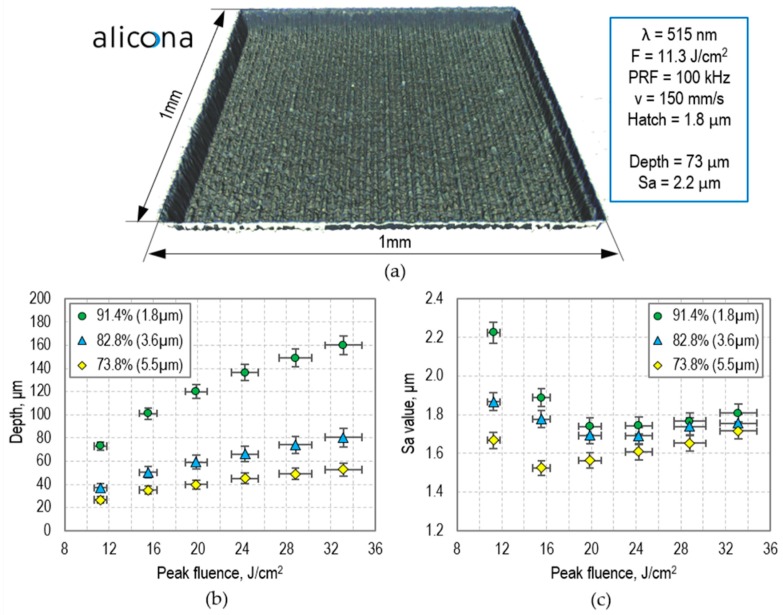
(**a**) Example of a 1 mm × 1 mm area generated by a moving 21-µm-diameter laser spot; (**b**) Ablation depth and (**c**) average surface roughness (Sa) of the 1 mm × 1 mm areas generated using a different combination of laser fluence and hatch distance. The laser machining was performed with PRF = 100 kHz and a scan velocity of 150 mm/s. Surfaces were measured using a 3D surface profilometer (Alicona InfinityFocus^®^).

Larger areas (including channels wider than 14 µm) can also be generated by the picosecond laser, typically by raster scanning the beam. The depth can be controlled by selecting an appropriate combination of laser fluence (F), pulse overlap (O), and hatch distance (i.e., a distance between the scanning lines). Figure 6a shows a 1 mm × 1 mm area that was generated using F = 11.3 µJ/cm^2^, O = 92.8% (PRF = 100 kHz and v = 150 mm/s), and hatch = 1.8 µm. The depth of this area is 73 µm, and the surface roughness (Sa) is 2.2 µm (as calculated for the central 0.8 mm × 0.8 mm region).

As can be seen in Figure 6b, the depth of the laser-machined areas is well controlled. If areas deeper than 160 µm are required, then the laser scan must be repeated. Using this approach, it is possible to generate very deep micro-wells, reservoirs, or even through holes (that can be used as inlet/outlet ports in glass covers for microfluidic devices). The surface roughness of the micro-wells and reservoirs was measured to be between 1.6 and 2.2 µm, as shown in Figure 6c. Finally, as can be noted from the example shown in Figure 6a, the laser-machined areas have inclined walls. The slope angle of these walls (α) was measured to be ~82°, which limits the aspect ratio of the laser-generated areas to 7 (calculated as tan(α)).

### 3.2. Calibration of the Laser Microwelding Process

The laser microwelding system enables the generation of weld seams at the interface of two glass plates, as demonstrated in References [41,42]. Previously, however, the welds were generated in relatively small areas (typically less than 5 mm × 5 mm). The challenge in this project was to generate welds along the glass–glass interface over a large area (75 mm × 25 mm). To ensure accurate positioning of weld seams during the laser microwelding process, it was necessary to take into account any tilt of the glass plates located in the holder. This was performed by recording the height position of the top glass plate at three different (X,Y) locations at which Fresnel reflection could be observed (see Reference [41] for more details). In this way, it was possible to compensate for the tilt and maintain the laser focus always at the same level (approximately 80 µm) below the glass–glass interface.

**Figure 7:**
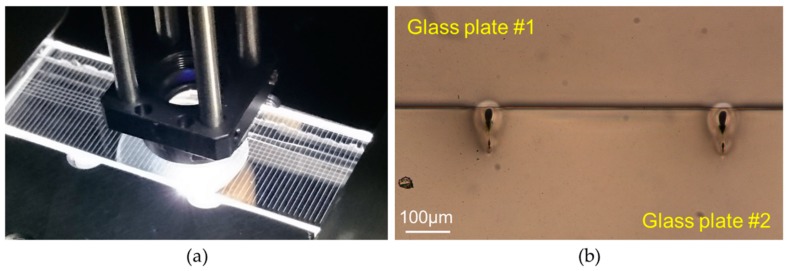
(**a**) Photograph taken during the laser welding of two 1.1-mm-thick glass plates; (**b**) cross-section of the weld seams generated at P = 2 W and v = 2 mm/s.

Figure 7a shows a photograph that was taken during the laser microwelding of two 1.1-mm-thick Borofloat^®^33 glass plates. The glass plates are blank, i.e., they do not contain any laser-generated patterns. The glowing lines seen in Figure 7a contain weld seams that were generated at the interface of the two glass plates. The cross-section of two weld seams is shown in Figure 7b, showing that they are teardrop shaped, and are 75 µm wide and 120 µm long. The welds were generated using an average laser power (P) of 2 W, moving the glass plates with a velocity of 2 mm/s. At this velocity, the pulse overlap was very high (nearly 99.98%).

The generation of weld seams occurs via multi-photon absorption and subsequent plasma generation inside the glass plates, as described in Reference [41]. By focusing the laser beam below the glass–glass interface, this plasma is generated in the top part of the lower glass (glass plate #2). This plasma expands and locally melts a small volume of surrounding glass that crosses into the bottom part of the cover glass (glass plate #1), and, when this solidifies on cooling, it forms a weld. Our previous work [42] showed that gaps smaller than 3 µm can be closed during the laser welding process.

### 3.3. Manufacturing of Microfluidic Devices

**Figure 8:**
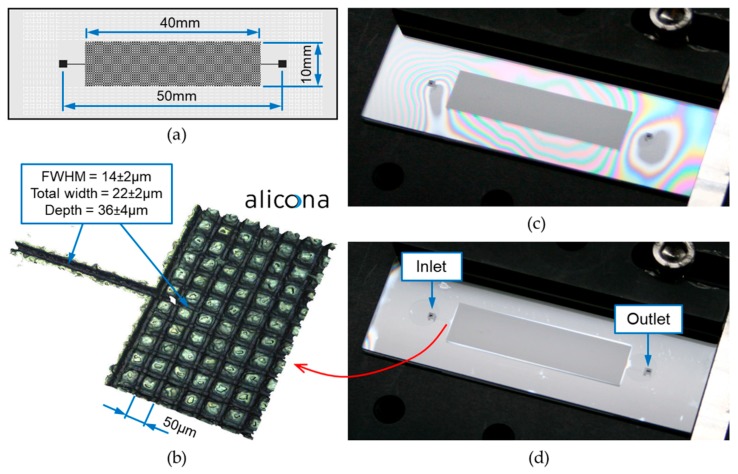
Example of laser-manufactured microfluidic device: (**a**) design; (**b**) 3D surface profile of its internal structure; (**c**,**d**) microfluidic device before and after laser microwelding, respectively.

Figure 8 shows a glass microfluidic device that was manufactured using the process described above. It contains a grid of microchannels that are 36 µm deep and 14 µm wide (FWHM). The distance between the microchannels is only 50 µm. The device also contains two 5-mm-long feed channels (also 36 µm deep and 14 µm wide) and two square inlet/outlet ports (1.5 mm × 1.5 mm).

The total time required for the generation of the microfluidic pattern and for the drilling of the inlet/outlet ports was approximately 15 min. Laser machining was carried out using three passes of the laser beam with PRF = 100 kHz and v = 150 mm/s. In this case, the laser fluence used had a constant value (F = 26.4 J/cm^2^) since a single depth was required. In order to drill two inlet/outlet ports (through holes), a total of nine passes were used. To maintain the laser beam focus during the drilling process, the sample was moved by a 0.1-mm distance toward the galvo-scanner after each laser scan.

**Figure 9:**
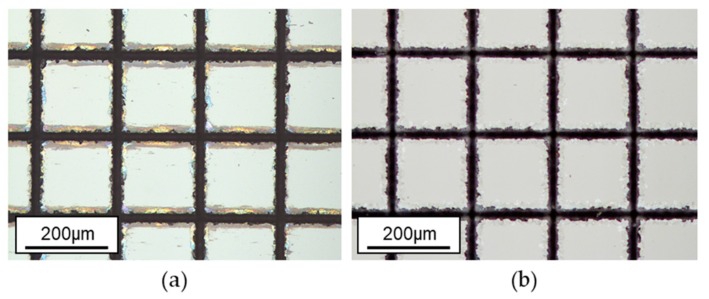
Optical microscope image of the laser-generated structure: (**a**) before and (**b**) after the cleaning in 5% hydrogen fluoride (HF) solution. The glass sample was etched for 2 min.

The laser-machined glass samples were cleaned in a bath containing 5% HF solution for two minutes in order to remove debris and redeposited material, as can be seen in Figure 9.

Figure 8c,d show the same microfluidic device before and after the laser microwelding process. Optical fringes visible in Figure 8c indicate a large gap between the two glass plates. Two adjacent bright or dark fringes represents a change in the gap length by λ/2.

Laser microwelding was started at the location where the gap was the smallest, i.e., near the inlet/outlet port on the right-hand side. In this way, it was possible to generate a plasma and initiate the process. Weld seams were generated around the microfluidic pattern, using the same laser parameters as those provided in Section 3.2. To ensure that the microfluidic device was properly sealed, weld seams were generated along several lines (separated by a 0.5-mm distance from each other) on all sides of the pattern. The location of the welds is shown in Figure 8a—see white dashes. This strategy allowed all existing gaps to be closed during the microwelding process, as can be seen in Figure 8d.

### 3.4. Fluid Flow Test

**Figure 10:**
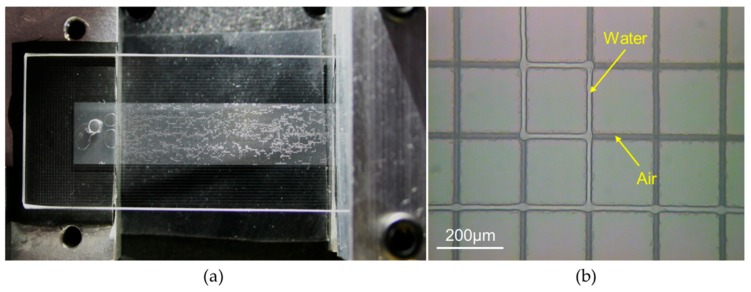
(**a**) Photograph taken during the testing of a laser-manufactured microfluidic device; (**b**) zoomed image of the microstructure partially filled in with water. This image was obtained using a Leica optical microscope.

The microfluidic channels were filled with DI water during its injection. Water did not leak from the devices, demonstrating that the laser-generated welds provided good sealing. To subsequently displace the water, nitrogen was injected into the devices under different pressures (up to 1.5 bar). These pressures did not cause physical damage to the devices. During the gas injection, nitrogen created preferential paths (so called “fingers”) to escape the device through the outlet port, as can be seen in Figure 10a. This phenomenon is called “viscous fingering” and can be observed in porous media (e.g., hydrocarbon-bearing rocks) when a less viscous fluid displaces a more viscous fluid [11,16,44].

Not all water could be removed from the microfluidic devices following the nitrogen injection. In some places, water was trapped in the microstructure, as shown in Figure 10b. This photo was taken following the fluid flow test when the inlet and outlet pressures were equal to the ambient pressure. The image shows a good contrast between water and air.

During the water injection at a pressure of ~1.5 bar, when the microfluidic device still contained air, it was observed that the pressure inside the device caused a little deformation of the glass plates, giving rise to a couple of optical fringes as a result of gap formation within the laser-machined area. Although the gap was very small (approximately 1 μm) and did not have any visible impact on the behavior of the flow of fluids, the authors consider this may be a problem in particular at high injection pressures, where injected fluids may start bypassing the microchannels by flowing through the gaps. One of the solutions to overcome this problem would be to use thicker (hence, stiffer) glass substrates for manufacturing the microfluidic devices. Another solution would be to place the microfluidic device inside a hermetic vessel and apply an external pressure onto the microfluidic device in order to compensate for the internal pressure. This solution requires a special vessel with a transparent window to provide optical access to the microfluidic device; however, it is feasible, as already shown in several publications [12,16,17,18,19]. Alternatively, it is of course possible to create additional weld regions in the interstices between microchannels, at the expense of increasing the manufacturing time.

## 4. Conclusions

This paper describes a laser-based process suitable for the rapid manufacturing of glass microfluidic devices. Using this process, it is possible to generate almost arbitrary enclosed microfluidic structures. The lateral resolution of the patterns, however, is limited to the laser spot size used for micromachining. The fluid flow tests performed for the microfluidic devices proved that a good sealing of the laser-generated microstructures can be obtained using the picosecond laser microwelding process. Weld seams generated by the laser not only eliminate any existing gaps between two glass plates, bringing the materials into close contact, but they also confine the flow of fluids to the designated areas. Hence, the laser microwelding process seems to be an attractive alternative to processes such as anodic bonding and thermal bonding.

Continuing work will focus on the investigation of the flow of different fluids through a range of pore network patterns generated by the laser. Currently, we are building a workstation dedicated to such experiments. The workstation will be equipped with a high-resolution camera with a high-zoom objective lens in order to observe the flow of fluids in microchannels, while fluids will be injected to the microfluidic devices at controlled flow rates using syringe pumps. In this way, it will be possible to investigate various fluid transport processes and to determine conditions at which injected fluids follow the microfluidic patterns, as well as identifying the limit beyond which fluids start flowing through small gaps between the glass plates, bypassing the microchannels.

Finally, it should be highlighted that the laser microwelding process has some limitations. One of the limitations is the lateral dimension of weld seams that determines the minimum size of the areas suitable for welding. In our case, two parallel microchannels cannot be isolated from each other by producing weld seams between them if the clearance between the microchannels is less than 100 μm. Weld seams of smaller dimensions, however, should be possible to generate using a different combination of laser welding parameters, i.e., using a different scan speed, pulse energy, repetition rate, and pulse duration. Unfortunately, the last two parameters cannot be changed in our laser system. In addition, equipping the laser microwelding system with a visualization system would simplify the necessary precise positioning of the welding samples. Using such a system, weld seams could be readily generated in specific locations, even in small areas between individual microchannels. Another limitation of our laser microwelding system is a welding speed (currently 2 mm/s). Higher welding speeds, however, can be achieved using ultrafast lasers operating at higher PRFs. For instance, using a laser with a PRF of 2 MHz, it should be possible to increase the welding speed to 10 mm/s, reducing the total welding time of microfluidic devices by a factor of five.

## Figures and Tables

**Table 1:** Laser beam diameters (2 ω_0_) and M^2^ values measured at the focal points. Measurements were performed using a scanning slit beam profiler (DataRay Beam-Map 2 sensor). The 2 ω_0_ values were measured at 1/e^2^ of the peak intensity. Output average power (P), pulse energy (E_P_), and peak fluence (F) calculated for each wavelength are also given here.

Wavelength (nm)	P (W)	Ep (μJ)	2 ω_0_ (μm)	M^2^ (value)	F (J/cm^2^) ^1^
1030	50	125	35 ± 1	1.3 ± 0.1	26.0 ± 1.5
515	30	75	21 ± 1	1.4 ± 0.1	36.3 ± 3.5
343	18	45	20 ± 1	2.1 ± 0.1	28.9 ± 2.9

^1^ Peak fluence was calculated as follows: F = 2 E_P_/(πω_0_^2^), where E_P_ is pulse energy and ω_0_ is the beam radius.

## Supplementary Materials

The following are available online at http://www.mdpi.com/2072-666X/9/8/409/s1, Table S1: Generation of narrow grooves on Borofloat^®^33 glass using PRF = 20 kHz. This dataset was used to plot graphs in Figure 5. Table S2: Generation of narrow grooves on Borofloat^®^33 glass using PRF = 10 kHz. Data not presented in the article. Table S3: Generation of 1 mm × 1 mm areas on Borofloat^®^33 glass. This dataset was used to plot graphs in Figure 6.
