# Supplementary material for: Rapid Laser Manufacturing of Microfluidic Devices from Glass Substrates
Source: Micromachines (Basel). 2018 Aug 17;9(8):409. doi: 10.3390/mi9080409 (PMC6187741; doi:10.3390/mi9080409)
Supplement: Supplementary file 1 [file micromachines-09-00409-s001.zip › micromachines-329291-supplementary.docx]

Supplementary Materials: Rapid Laser Manufacturing of Microfluidic Devices from Glass Substrates

**Table S1.** Generation of narrow grooves on Borofloat^®^33 glass using PRF = 20 kHz. This dataset was used to plot graphs in Figure 5.

| Ep (μJ) | F (J/cm^2^) | F_max_ (J/cm^2^) | F_min_ (J/cm^2^) | 1 Pass | | 2 Passes | | 3 Passes | | 4 Passes | | 5 Passes | |
| --- | --- | --- | --- | --- | --- | --- | --- | --- | --- | --- | --- | --- | --- |
|  |  |  |  | **Depth (μm)** | **Width (μm)** | **Depth (μm)** | **Width (μm)** | **Depth (μm)** | **Width (μm)** | **Depth (μm)** | **Width (μm)** | **Depth (μm)** | **Width (μm)** |
| 57.4 | 33.1 | 34.8 | 31.6 | 8.1 | 17.0 | 17.1 | 15.6 | 23.7 | 15.1 | 29.9 | 14.6 | 36.4 | 15.0 |
| 49.9 | 28.8 | 30.2 | 27.5 | 7.4 | 14.3 | 16.8 | 13.8 | 23.1 | 13.1 | 28.9 | 12.8 | 34.0 | 12.3 |
| 42.0 | 24.3 | 25.4 | 23.1 | 7.4 | 14.4 | 14.8 | 14.1 | 20.9 | 13.6 | 25.4 | 13.5 | 30.1 | 12.5 |
| 34.4 | 19.9 | 20.8 | 19.0 | 8.2 | 14.4 | 13.5 | 13.9 | 18.5 | 12.0 | 24.1 | 12.8 | 29.7 | 12.8 |
| 26.9 | 15.5 | 16.3 | 14.8 | 7.2 | 15.2 | 12.6 | 12.2 | 15.7 | 13.9 | 21.5 | 11.6 | 24.7 | 11.3 |
| 19.5 | 11.3 | 11.8 | 10.7 | 6.0 | 13.8 | 11.3 | 14.9 | 14.4 | 13.3 | 18.4 | 11.1 | 20.6 | 11.8 |

Common parameters: λ = 515 nm; PRF = 20 kHz; v = 40 mm/s; ω = 10.5 μm (beam radius); Pulse overlap = 90.48% (2 μm pulse-to-pulse spacing).

**Table S2.** Generation of narrow grooves on Borofloat^®^33 glass using PRF = 10 kHz. Data not presented in the article.

| Ep (μJ) | F (J/cm^2^) | F_max_ (J/cm^2^) | F_min_ (J/cm^2^) | 1 Pass | | 2 Passes | | 3 Passes | | 4 Passes | |
| --- | --- | --- | --- | --- | --- | --- | --- | --- | --- | --- | --- |
|  |  |  |  | **Depth (μm)** | **Width (μm)** | **Depth (μm)** | **Width (μm)** | **Depth (μm)** | **Width (μm)** | **Depth (μm)** | **Width (μm)** |
| 57.4 | 33.1 | 34.8 | 31.6 | 8.7 | 15.5 | 15.0 | 13.1 | 22.6 | 12.0 | 30.2 | 14.0 |
| 49.9 | 28.8 | 30.2 | 27.5 | 8.3 | 14.2 | 15.3 | 13.8 | 20.5 | 13.9 | 28.1 | 12.6 |
| 42.0 | 24.3 | 25.4 | 23.1 | 9.3 | 14.4 | 14.1 | 13.4 | 21.0 | 12.6 | 25.7 | 11.3 |
| 34.4 | 19.9 | 20.8 | 19.0 | 8.7 | 11.9 | 14.5 | 13.0 | 19.2 | 12.6 | 23.4 | 11.0 |
| 26.9 | 15.5 | 16.3 | 14.8 | 6.9 | 13.5 | 13.7 | 12.0 | 17.4 | 11.5 | 21.3 | 11.5 |
| 19.5 | 11.3 | 11.8 | 10.7 | 6.9 | 15.5 | 11.4 | 11.7 | 13.6 | 12.3 | 17.7 | 10.4 |

Common parameters: λ = 515 nm; PRF = 10 kHz; v = 20 mm/s; ω = 10.5 μm (beam radius); Pulse overlap = 90.48% (2 μm pulse-to-pulse spacing).

**Table S3.** Generation of 1 mm × 1 mm areas on Borofloat^®^33 glass. This dataset was used to plot graphs in Figure 6.

| Ep (μJ) | F (J/cm^2^) | F_max_ (J/cm^2^) | F_min_ (J/cm^2^) | Hatch = 1.8 μm | | | Hatch = 3.6 μm | | | Hatch = 5.5 μm | | |
| --- | --- | --- | --- | --- | --- | --- | --- | --- | --- | --- | --- | --- |
|  |  |  |  | **Depth (μm)** | **Sa (μm)** | **Sq (μm)** | **Depth (μm)** | **Sa (μm)** | **Sq (μm)** | **Depth (μm)** | **Sa (μm)** | **Sq (μm)** |
| 57.4 | 33.1 | 34.8 | 31.6 | 160 | 1.81 | 2.32 | 80 | 1.76 | 2.24 | 53 | 1.72 | 2.17 |
| 49.9 | 28.8 | 30.2 | 27.5 | 149 | 1.77 | 2.24 | 74 | 1.74 | 2.21 | 49 | 1.65 | 2.09 |
| 42.0 | 24.3 | 25.4 | 23.1 | 136 | 1.74 | 2.22 | 66 | 1.69 | 2.14 | 45 | 1.61 | 2.03 |
| 34.4 | 19.9 | 20.8 | 19.0 | 120 | 1.74 | 2.24 | 59 | 1.69 | 2.16 | 40 | 1.56 | 1.97 |
| 26.9 | 15.5 | 16.3 | 14.8 | 101 | 1.89 | 2.45 | 50 | 1.78 | 2.28 | 35 | 1.52 | 1.94 |
| 19.5 | 11.3 | 11.8 | 10.7 | 73 | 2.23 | 2.84 | 37 | 1.87 | 2.35 | 27 | 1.67 | 2.12 |

Common parameters: λ = 515 nm; PRF = 100 kHz; v = 150 mm/s; ω = 10.5 μm (beam radius); Pulse overlap = 92.9% (1.5 μm pulse-to-pulse spacing).
